# A Cost-Benefit Analysis of the Official Development Assistance Project on Maternal and Child Health in Kwango, DR Congo

**DOI:** 10.3390/ijerph15071420

**Published:** 2018-07-05

**Authors:** Changwoo Shon, Tae Ho Lee, Grace Ossak Ndombi, Eun Woo Nam

**Affiliations:** 1 Department of Urban Society, The Seoul Institute, 57 Nambusunhwan-ro, 340-gil, Seocho-gu, Seoul 06756, Korea; cwshon@si.re.kr; 2 Yonsei Global Health Center, Yonsei University, Wonju, Gangwon-do 26493, Korea; leetaeh5@naver.com (T.H.L.); ndombiossak@hotmail.com (G.O.N.); 3 Department of Health Administration, Graduate School, Yonsei University, Wonju, Gangwon-do 26493, Korea

**Keywords:** economic evaluation, Cost-Benefit Analysis (CBA), Official Development Assistance (ODA), maternal and child health, maternal mortality, infant mortality, under-five mortality

## Abstract

A project on maternal and child health (MCH) was conducted by the Korea International Cooperation Agency to reduce maternal and child mortality rates in Kwango, Democratic Republic of Congo (DRC). The objective of this study was to evaluate the costs and benefits of the MCH project, which was under Official Development Assistance for a period of 3 years from 2014 to 2016. The study conducted a cost-benefit analysis (CBA) using a benefit-cost ratio (BCR). The costs were the total costs incurred in implementing the MCH project. The benefits of the MCH project were estimated as the monetary values of the reduction in maternal mortality rates and the mortality rates of infants and children aged under 5 years. The adjusted costs that converted the time value for 2016 were estimated as USD 1,969,532 as part of the CBA. The benefits of reduced maternal mortality and the mortality of infants and children aged under 5 years were estimated as USD 681,416, USD 4,332,376, and USD 1,710,184, respectively, in monetary terms. The total benefits were estimated as USD 6,723,976 and the BCR was calculated at 3.41. In addition, the benefits were estimated by the different economic assumptions through a sensitivity analysis. The MCH project was economically satisfied under the most conservative assumptions.

## 1. Introduction

Maternal health is at the core of public health in that prioritizing it can prevent diseases and disabilities among mothers and children well in advance, especially when the policy targets pertain to developing countries. Not only does good health for mothers and children lead to good health for families, but it also affects children’s education and growth in productivity in the long-term [1]. The World Health Organization (WHO) emphasized the importance of maternal and child health (MCH) through Millennium Development Goals (MDGs) 4 and 5. Since the 1990s, the rate of 41% of women who died during pregnancy or childbirth in sub-Saharan Africa has notably decreased [2]. Still, approximately 287,000 women have died and about 5.7 million people have suffered from illnesses or disabilities due to complications during pregnancy or childbirth in 2010. Against this backdrop, the project for capacity building on maternal, newborn, and child health care in the Kwango provincial division of health in the Democratic Republic of Congo (DRC) was carried out by the Korea International Cooperation Agency (KOICA) as a grant aid project at the request of the DRC government. The objective of the MCH project by KOICA was to reduce maternal and child mortality rates in the Kenge and Boko health zones, which are located in Kwango. The MCH project under Official Development Assistance (ODA) has been the responsibility of the Yonsei Global Health Center (YGHC) for 3 years since 2014. The MCH project was based on the three-delay model to reduce the number of maternal and child deaths. The three-delay model is an integrated approach to address each of the three phases of delay issues that women and children face while trying to access safe childbirth [3]. The three delay issues are: (1) delay in decision to seek care; (2) delay in arrival at health facilities; and (3) delay in the provision of adequate care. Therefore, the MCH project was composed of various activities to resolve each phase through: (1) enhancement of awareness among communities; (2) improvement of accessibility of to healthcare services; and (3) improvement of the quality of healthcare services. Improving access to maternal and child health services by minimizing the three delays has the potential to reduce mortality [4].

Meanwhile, ODA is defined as government aid designed to promote the economic development and welfare of developing countries [5]. If ODA is provided to developing countries, it is necessary to conduct an evaluation of programs or services. Evaluation is one of the ways in which funders and policy-makers can confirm the performance and effectiveness of ODA programs. According to the Organization for Economic Cooperation and Development, Development Assistance Committee (OECD-DAC), the evaluation of an ODA project is defined as the systematic and objective assessment of an on-going or completed project, program, or policy, its design, implementation, and results. In addition, ODA evaluation can not only improve future assistance policy, programs, and projects through feedback on lessons learned but also provide a basis for accountability, including the provision of information to the public [6]. For ODA evaluation, we considered the DAC’s five evaluation criteria, consisting of relevance, effectiveness, efficiency, impact, and sustainability. An intuitive and effective evaluation technique is the economic appraisal, which serves to determine the appropriateness of the implementation of a project by comparing monetary values. However, it has not been long since economic evaluation was introduced in the ODA field. Since the global economic crisis in the early 2000s, economic evaluation has been introduced as a matter of interest on part of donor countries in furthering efficiency, and the transparency of resource allocation has increased [7,8]. The E.U. has also emphasized the introduction of economic assessments as one of the ways to curb corruption in developing countries [9].

In terms of economic evaluation, there are three appraisal techniques in health promotion programs, namely cost-benefit analysis (CBA), cost-effectiveness analysis (CEA), and cost-utility analysis (CUA). The feature that distinguishes these three techniques from each other is the way in which the consequences of health programs are evaluated. CBA evaluates the consequences of the programs in monetary units, thereby enabling a decision-maker to make a direct comparison of the program’s incremental costs with its incremental benefits in commensurate units of measurement. CBA was performed in this study since it is more intuitive and more persuasive than are the other two techniques [10]. Although ODA evaluation using economic appraisal has these advantages, only a few studies have evaluated the cost-effectiveness of health promotion programs provided to mothers and children as ODA projects. Thus, this study was performed to analyze the costs and benefits of MCH programs during the period between 2014 and 2016 in Kwango, DRC.

## 2. Materials and Methods

### 2.1. CBA Conceptualization and Method

As a matter of health security in families, the MCH project is expected to increase quality of life. This is instrumental in the attainment of long-term benefits, such as social-emotional functions, health behaviors, and economic self-sufficiency. Therefore, the total economic benefits of the MCH project would be expected to be relatively large. However, the focus of effectiveness in this study was limited to maternal health and reduction of mortality rates owing to the project budget and in order to estimate the effect conservatively. We estimated the present value of program benefits in 2016, in U.S. dollars, under three main categories: (a) reductions in the number of maternal deaths due to the delivery of facilities, (b) reductions in the number of infant deaths due to antenatal and postnatal care, and (c) reductions in the number of deaths among children under age 5 due to the implementation of the nutrition program. Finally, the benefit-cost ratio (BCR) suggested at the end of the study was calculated based on the expected benefits of the MCH project. In addition, we tested the validity of economic costs and benefits for different model assumptions as a sensitivity analysis using a tornado diagram. A tornado diagram is a useful way to visualize the sensitivity of a result to changes in economic assumption.

### 2.2. MCH Project Costs

Costs related to the MCH project were estimated based on the budget statement provided by the KOICA. Almost all of the direct costs incurred in a maternal and child health project, such as doctors’ and nurses’ expenses, education costs, and other program costs, are included in the budget statement. In order to make more accurate estimates, indirect costs, such as time cost of mothers, transportation cost, and informal care cost, should be estimated as well as the direct cost based on the budget statement, but it could not be included in the analysis due to the limitation of valid and representative data acquisition. Nevertheless, cost estimates based on budget statements have the advantage of being relatively free of measurement errors that occur as a consequence of the provided data differing from the real numbers. The total costs consisted of nine items, including costs of (1) Capacity building (training of community people, health professionals, midwives, and community health workers), (2) Support for the Maternal and Newborn Child Healthcare Unit (MNU), (3) Promotion of MCH services, (4) Support for nutrition management services (Training on nutritional management for health professionals and community health workers and supply and procurement of nutrition materials), (5) Monitoring and evaluation, (6) Financial support for monitoring and supervision of the Ministry of Public Health (MoPH), (7) Dispatch of health experts, (8) Local cost of project management (Local office and local staff and promotion of project), and (9) Expenditure incurred by KOICA. The expenditure incurred by KOICA included medical devices and materials and the construction of community health centers, hospitals, and the MNU. All costs measured were converted into U.S. dollars using the dollar exchange rate for Korean Won for each year (1 USD = 1053.2 for 2014, 1131.5 for 2015, and 1160.5 for 2016) [11]. In addition, we discounted the project cost because the value of a unit of consumption to individuals and society decreases over time. We calculated future values for 2016 using the WHO-recommended social interest rate (3%) for comparability across studies [12].

The following is the equation for Future Value:

Future Value (FV) = Present Value (PV) × (1 + *r*) *^n^* (1)

where *r* is the interest rate.

### 2.3. MCH Project Benefits

The direct effects of the MCH project are, in the short-term, a reduction of maternal death rates and death rates among infants and children under age 5 and strengthening of maternal capacity, and, in the long-term, a rise in Gross Domestic Product (GDP) at the macro-level [13]. The estimates of the benefits in this study excluded the secondary benefits of productivity improvement but included three kinds of benefits imported from past studies, namely maternal mortality, infant mortality, and mortality among children under age 5 [14,15]. In general, it is an appropriate way to estimate the effects based on the actual number of survivors and deaths, but the local monitoring system was not well-established enough to produce accurate statistics in the DRC. Therefore, the effects were estimated by referring the values of the previous studies whose effects were verified [16]. Total benefits were estimated by multiplying the number of survivors due to intervention and the GDP per capita up to the expected life expectancy. If the social discount rate is derived from the aggregation of individual discount rates and the distribution of discount rates across the population, it can be smaller than the average of the individual rates and will decline over time [17]. However, a declining rate of discount has not commonly been used in health-related CBA [12]. For comparison with previous studies, the term structure of interest rate assumed a fixed interest rate during the benefit periods and the WHO-recommended social discount rate (3%) was applied [12].

### 2.4. Benfit-Cost Ratio (BCR) and Net Present Value (NPV) of MCH Project

Using the estimated costs and estimates for 3 years of the MCH, the BCR was calculated. Generally, the BCR is a useful criterion in project investment and is defined as the present value of net positive cash flow divided by net negative cash flow at a certain point in time. If the BCR is greater than 1, the program implemented is considered economically effective [10]. The results of the sensitivity analysis were presented through a tornado diagram with the BCR of various economic conditions.

The following is the equation for the BCR:

BCR = PV of Net Positive Cash Flow/PV of Net Negative Cash Flow
(2)

In addition to the BCR, the NPV, which is the difference between the present value of cash inflows and the present value of cash outflows over a period of time, was calculated. Generally, a positive NPV means that the project will be profitable, and a negative NPV means that it will result in a net loss.

The following is the equation for NPV:

(3) $$\mathrm{NPV}=\overset{T}{\underset{t=1}{\sum }}\frac{C_{t}}{{\left(1+r\right)}^{t}}-C_{o}$$

where *C_t_* is the net cash inflow during the period *t*; *C_o_* is the total initial investment cost; *r* is the discount rate; and *t* is the number of time periods.

## 3. Results

### 3.1. Cost Estimates

This study included all costs incurred in implementing the MCH project in the DRC (Table 1). The costs of implementing the MCH project in the DRC have been shown for 3 years from 2014 to 2016. Costs were categorized into capacity building costs, MNU costs, MCH promotion costs, nutrition management services costs, monitoring and evaluation costs, financial support for the monitoring and supervision of the MoPH, costs incurred in the dispatch of health experts, project management costs, and expenditure incurred by KOICA. Capacity building costs included the costs incurred in training people in the community, health professionals, and community health workers. MNU costs included support for the implementation of the MNU. The costs incurred in the MCH promotion also included costs incurred in the promotion of the project and activities as part of the project through radio broadcasting and signboards. The costs incurred on nutrition management services included costs incurred in training for health professionals and community health workers on nutrition management as well as the procurement and supply of nutrition materials. Monitoring and evaluation costs included costs incurred on periodic surveys and support for the monitoring of the project. Financial support for monitoring and supervision included costs according to the hierarchical levels of the MoPH. The costs incurred on the dispatch of health experts included expenditure on dispatching the project manager and junior consultants. Project management costs included expenditure on operating a local office and hiring local staff as well as promoting the project. The expenditure incurred by KOICA included the construction costs for a new MNU and healthcare facilities as well as medical supplies.

In summary, the costs incurred in capacity building, MCH promotion, nutrition management services, monitoring and evaluation, the dispatch of health experts and project management, and a part of the infrastructure were all selected for the estimation of the total costs. In the case of infrastructure, the components included for cost estimations were the MNU, new construction and remodeling of health facilities (i.e., a health center and a secondary hospital), and the provision of medical equipment. However, financial support for the monitoring and supervision of the MoPH and the expenditure incurred by KOICA (excluding infrastructure) was not considered.

The costs incurred for this project were USD 550,001 in 2014, USD 718,074 in 2015, and USD 646,420 in 2016. For the cost-benefit analysis (CBA), the total cost, after adjusting for the time value of money, was estimated as USD 1,969,532 (Table 2).

### 3.2. Benefit Estimates

The main contents of the MCH project are prenatal and postpartum care and delivery by health workers (i.e., Medical doctors and nurses). Midwives do not work at project areas because of an insufficiency in manpower. Nutrition programs for children under age 5 were also included. Through these programs, it is expected that maternal mortality rates, and the mortality rates of infants and children under age 5, will reduce in the short-term. In the mid-term and in the long-term, it is expected that the national economy will grow with the increase in population. Therefore, the benefits of the MCH project estimated include an increase in lifetime earnings due to the reduction in mortality rates. Specifically, the benefits were estimated based on the differences between the expected number of deaths when the program was not provided and the expected number of deaths due to the implementation of the program.

From 2014 to 2016, the number of people who were benefited by these programs is presented category-wise as follows. The number of women whose children were delivered by health workers in the facilities was 14,196. The number of women using antenatal care programs was 15,895. The number of children under the age of 5 using nutrition programs was 3049. In the case of prenatal care, we conducted a total of two surveys since the project was implemented to confirm the number of women in terms of continuity of care. An average of 4.53 women in the first year of the study and 3.92 women in the second year of the study benefited from the services, which means that most women received four or more antenatal care visits, which is the number of visits that is recommended [18].

Nutrition programs consisted of four focus areas, namely health education, measurement and procurement of nutrition, registration and monitoring of malnutrition, and promotion of nutrition programs. Health education was conducted for chief nurses and community health workers in the project area, focusing on screening, treatment, and monitoring of malnutrition. When it came to the measurement and procurement of nutrition, we assessed the comprehensive status of malnutrition among children by measuring the mid-upper arm circumference (MUAC), weight, and height. Nutrition supplies were provided according to the measured extent of malnutrition among the children. Registration and monitoring of malnutrition data were managed by using the form provided by YGHC. The form consisted two parts, namely general information on the child, such as name, height, weight, age, MUAC, and occurrence of edema and/or diarrhea, and information on nutrition provided as well as the malnutrition status. The promotion of nutrition programs was conveyed by radio broadcast and focused on addressing malnutrition among children aged under 5 years. Antenatal care and care delivered by health workers in the facilities were the integrated results of programs such as capacity building, support for the MNU, promotion of the MCH service, and financial support for the monitoring and supervision of the MoPH. The beneficiaries of this project were women living in the project area all within the reproductive age range. The number of services provided, such as prenatal care or those delivered by health workers in the facilities, was reported by the chief nurses in each health center by filing a monthly report under the Système National d’Information Sanitaire (SNIS), which is a national record of health information. We calculated and estimated the number of women who received services using data from the monthly reports and the results of our survey.

As of 2015, in the DRC, the maternal mortality rate was 693 per 100,000 [19], the infant mortality rate was 74.5 per 1000, and the mortality rate among children under age 5 was 98 per 1000 [20]. Based on this, the number of women who died from pregnancy-related or childbirth-related complications was 98 in the Kenge and Boko areas. The number of deaths among infants and children aged under 5 years was 1184 and 328, respectively, in the same areas. The extent of reduction in the mortality rates was estimated based on the results of past studies.

The most effective way of reducing maternal mortality is known as facility delivery. It increases the survival rate and reduces the number of maternal and newborn deaths by ensuring safe birth and preventing complications [21,22]. In this context, we assumed that the maternal mortality rate was reduced by 69% as a result of facilitating delivery after the interventions using the values given in the systematic review paper evaluating 70 journals dealing with maternal mortality reduction in sub-Saharan Africa [23]. The average age of pregnant women in this study that was used for research was 27.5 years, which was derived from the data in a maternal health study situated in Ethiopia [24], a neighboring country with similar life expectancy levels and birth rates. The reduction in infant mortality rates by 28%, which was suggested by the aforementioned study, was used in order to estimate the effect of antenatal care. Finally, as suggested by the WHO, it was estimated that the nutrition program would have reduced the mortality rate of children under 5 years by 45% [25]. Based on these statistics, we assumed that the benefits were intended for women for 34 years, for 59 years for infants, and for 55 years for children aged under 5 years taking into account age and life expectancy (59.8 years) as of 2016. The benefits calculated for one person for one year were regarded as GDP per capita (USD 461.2) in 2016 [26], and then we estimated future benefits by using a social discount rate of 3%.

Table 3 shows the benefit estimation for the MCH project from 2014 to 2016. The benefit estimation after the implementation of the MCH project is $6,723,976. The monetary value that the surviving women will earn is $681,416, the monetary value that the surviving infants will earn is $4,332,376, and the monetary value that children aged under 5 years will earn is $1,710,184. All these amounts were calculated taking into account the number of survivors in all the categories, the GDP rates, and the benefits accrued.

### 3.3. Cost-Benefit Estimates with BCR and NPV

Based on the cost and benefit of the project, we attempted to identify the relationship between the cost and the benefits of the MCH project by calculating the BCR. It is a useful criterion in studying project investment and is defined as the present value of net positive cash flow divided by the net negative cash flow. In this study, the economic benefit derived from this MCH project was USD 6,723,976 and the total cost was USD 1,969,532 (Table 4). Therefore, the BCR was 3.41, which means that the MCH project was more than 3 times more likely to provide benefits than costs. Meanwhile, the NPV was USD 4,754,411, with a value greater than zero, confirming that the project was profitable.

### 3.4. Sensitivity Analysis

**Figure 1:**
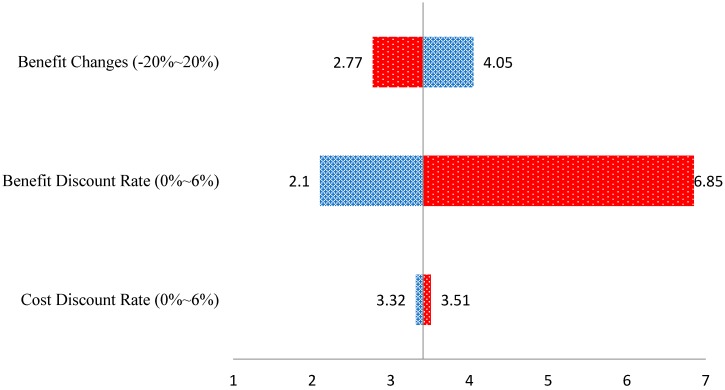
The changes of BCRs from the sensitivity analysis.

Generally, the reliability and the comparability of the results of a study can be guaranteed if an economic evaluation is performed using a social discount rate. In addition, to increase the robustness of the results, sensitivity analysis using “one-way analysis” was performed. With one-way analysis, each uncertain component of an evaluation changes individually, while another component maintains a basic case specification [27]. According to previous studies and WHO recommendations [12,28], a discount rate of 3% is appropriate for the cost for the base case, and it is appropriate to change the discount rate up to 6% when performing a sensitivity analysis. Also, a discount rate of 3% for the benefit can be reduced to 0% when conducting a sensitivity analysis. In addition, we assumed that the benefit of the MCH project could change from −20% to +20%. With reference to these guidelines, a sensitivity analysis was conducted by changing the discount rates for the cost and benefit from 0% to 6% and the benefit changes from −20% to 20%. The BCR for each economic assumption was calculated and a range of the BCR was presented through tornado diagrams. As a result, assuming that the benefit discount rate is 0%, the BCR rises to 6.85, but assuming the benefit change to be −20%, the BCR is lowered to 2.77. On the other hand, the cost discount rate had a relatively small impact on the BCR even under various assumptions. In conclusion, we confirmed that estimated benefits exceeded estimated costs even under the most conservative assumptions (Figure 1).

## 4. Discussion

The MCH project was designed and performed as an integrated project based on a three-delay model to control the three delay issues on maternal and child mortality [3]. This study was conducted to evaluate the MCH project using CBA methods. The results of the study are summarized as follows. First, the results of the CBA in the efficiency evaluation estimated that 68 maternal deaths, 332 infant deaths, and 148 deaths among children aged under 5 years were avoided annually as a result of the MCH project in Kwango, DRC. Second, the estimated cost was USD 1,969,532 and the economic benefits of this project amounted to USD 6,723,976. The BCR based on the CBA of the MCH project was 3.41 and the NPV was USD 4,754,444; so, the benefits outweigh the cost. Therefore, the MCH project is economically satisfactory. However, the BCR of the study, which was relatively lower than the result (BCR = 11) derived from the Maternal and Child Health Project conducted in Nigeria by the Agency for International Development (USAID) in 2010 [29]. Nevertheless, maternal and child health project is still cost-effective for most African countries where health and medical care infrastructure is not well-designed. Decision-makers of the DR Congo would identify that the maternal health project with the high BCR is cost-effective [30].

The reasons why the program was successfully implemented are as follows. The MCH project was successful in that it aimed at cooperating and controlling each program to ensure and receive quality healthcare services. An integrated approach is important to design and operate an effective and efficient MCH project. In addition, an effective project designed by YGHC, which has diverse experience in global health research in developing countries, has helped achieve the positive outcome. They hired professional workers to manage, implement, and monitor the project. The operation team consisted of experts in community nursing, health administration, and MCH for the project. Further, we also collaborated with professionals from the School of Public Health, Kinshasa University, for the measurement of performance in the project and we supported community health workers and community leaders toward active management and monitoring beneficiaries of the project. Lastly, for sustainable management and monitoring, this project had cooperative relationships with the MoPH and its affiliated organizations in the DRC and operated according to the national healthcare system.

Despite these achievements, there are still many challenges to improving maternal health in the DRC, which is a representative low-income country in sub-Saharan Africa. As of 2017, the per capita GDP (PPP) for the DRC was USD 800, the life expectancy was 57.7 years, and the number of doctors per 1000 people was only 0.09 [31]. Countries in sub-Saharan Africa with higher maternal and infant mortality rates, including the DRC, face problems in the form of a lack of access to resources for MCH care, a lack of staff to care for them, and huge burdens of cost while receiving services [32]. In other words, the DRC still faces a difficult challenge in training and allocating appropriate health care workers to increase the sustainability of MCH projects in the future. Except for hospitals with doctors in rural areas, and at the project site, almost all nurses work as birth assistants without any background as midwives or midwifery education. Midwives need to improve maternal and newborn survival rates in developing countries. However, it is so difficult to become educated because midwives do not have enough money to afford training. Therefore, it is necessary for the national government to support regular and supplementary education for midwives in general and provide quality midwifery education in rural areas. Even if health professionals are trained midwives, they tend to move to bigger cities for jobs at health care centers that offer them better wages than centers in rural areas. Therefore, it is necessary to establish a legal basis for mandatory or minimum staffing in rural hospitals for certain periods of time. The health system in the DRC has suffered many difficulties for many years as a result of the lack of investment. What makes the matter worse are the recent wars in the country [33]. Thus, it is known that more than one million people do not receive clean drinking water, food, and health care services.

There are some limitations to this study, although the results are meaningful to developing countries. First, in the process of estimating the benefits of the project, we could not to take into account the actual number of deaths and surviving individuals. It was a realistic constraint to follow up after the end of the project period. We tried to investigate the number of actual survivors and deaths, but the local monitoring system did not work properly. For example, it was difficult to guarantee the reliability of the follow-up data because some subjects were duplicated, some of the deaths were recorded as survivors, and some of the survivors were recorded as deaths. In fact, not only in the DRC but in other sub-Saharan countries it is very difficult to obtain accurate statistics on the number of deaths after an ODA project has been implemented due to the lack of infrastructure, including a vital registration system [16]. Thus, we had no choice but to estimate the benefits based on the results of past studies targeting countries with DRC-like health status and healthcare infrastructures. For a more accurate assessment of ODA projects in the future, it is necessary to consider the follow-up process after a program has been provided when planning the contents and budget of the ODA project. Second, this study was confined to only some parts of the DRC. Therefore, it may be difficult to generalize that the MCH project would have the same effect in other countries in Africa. Despite the limitations, it is significant that this study emphasized the importance of maternal health and suggested prioritizing MCH in sub-Saharan Africa by estimating the economic benefits of projects focusing on the area.

This study focused on the direct effects of improving maternal and newborn health through the ODA project and suggested the economic effectiveness of maternal health care. Based on the results of this study, efforts should be made to encourage donors to contribute and raise awareness on the state of maternal health among policy-makers in sub-Saharan countries as well as among the staff in the DRC government. It should also be emphasized that maternal health contributes to poverty reduction in the long-term, and it is important to focus on maternal health rather than simply providing services, thereby improving women’s health through empowerment. Beyond the direct effects of the maternal health program, we hope that research on the indirect and long-term effects of maternal health, such as education, industry, and economy, will be carried out in the future.

## 5. Conclusions

Maternal health is essential for good health in children and families. Furthermore, a mother plays a key role in care-giving when it comes to handling nutrition and health behaviors. However, a lot of mothers suffer from a lack of health-related resources and bear the brunt of poor conditions in sub-Saharan countries. We emphasized that maternal and infant mortality rates can be decreased significantly through better facilities for delivery and prenatal and postnatal care. The mortality rate for children under the age of 5 can also be reduced by implementing nutrition programs and ensuring that the economic benefits of all projects are satisfied. Conclusively, in this study, the economic benefit derived from the KOICA-YONSEI MCH project was USD 6,723,976 and the total costs incurred amounted to USD 1,969,532. Therefore, the BCR and the NPV were calculated as 3.41 and USD 4,754,444, respectively.

The cost-benefit study can be used not only for donor countries but also for decision-makers in the recipient country as a basis for decision-making. Although it is important to provide and evaluate effective programs, it should be noted that, ultimately, the capacity of recipient countries should be strengthened so that they can have their own resources and systems.

## Figures and Tables

**Table 1:** Intervention costs of the maternal and child health (MCH) project in the Democratic Republic of Congo (DRC) by year (Unit: USD).

Division	2014		2015		2016	
	Budget	Costs	Budget	Costs	Budget	Costs
1. Capacity Building	102,909	102,909	167,377	61,387	339,548	175,676
1.1. Training of community people	-	-	1569	-	11,534	11,534
1.2. Training of HP (Child health)	102,909	102,909	-	-	-	-
1.3. Training of HP (Maternal health)	-	-	61,387	61,387	164,142	164,142
1.4. Training of CHW	-	-	104,421	-	136,871	-
2. Support for MNU	-	-	-	-	1947	-
3. Promotion of MCH service	16,363	16,363	9088	9088	3028	3028
4. Support for nutrition management service	47,959	47,959	282,745	282,745	22,933	22,933
5. Monitoring and evaluation	46,824	46,824	42,599	42,599	59,759	59,759
6. Financial Support for monitoring and supervision of MoPH	60,202	-	30,275	-	77,592	-
7. Dispatch of health experts	239,631	239,631	177,135	177,135	165,872	165,872
8. Project management	96,315	96,315	145,121	145,121	138,071	138,071
9. KOICA’s expenditure	-	-	-	-	2,432,426	81,081
Total costs	610,203	550,001	854,340	718,074	3,241,176	646,420

Abbreviations: HP, Health Professional; CHW, Community Health Worker; MNU, Maternal and Newborn Child Healthcare Unit; MoPH, Ministry of Public Health; KOICA, Korea International Cooperation Agency.

**Table 2:** Present value of intervention costs of the MCH project in the DRC.

Division	2014	2015	2016	Total
Total nominal costs (USD)	550,001	718,074	646,420	1,914,495
Present value of 2016 (USD)	583,496	739,616	646,420	1,969,532

**Table 3:** Benefit estimation for the MCH project (2014–2016).

Division	The Number of Survivors	Benefit Period	Monetary Value	Total Monetary Value
Benefit of reduced MMR	68	34	$681,416	$6,723,976
Benefit of reduced IMR	332	59	$4,332,376	
Benefit of reduced U5MR	148	55	$1,710,184	

Abbreviations: MMR, Maternal Mortality Rate; IMR, Infant Mortality Rate; U5MR, Under 5 Mortality Rate.

**Table 4:** The results of the cost-benefit analysis (CBA) for the MCH project.

Division	Total Benefits	Total Costs	BCR	NPV
MCH Project	$6,723,976	$1,969,532	3.41	$4,754,444

Abbreviations: BCR, benefit-cost ratio; NPV, net present value.
